# Dispersion‐Engineered Broadband Transparent Meta‐Cloak

**DOI:** 10.1002/nap2.70129

**Published:** 2026-05-12

**Authors:** Dan Liu, Xingshuo Cui, Chunsheng Guan, Xiaochun Liu, Wenye Ji, Fei Gao, Tong Cai

**Affiliations:** ^1^ State Key Laboratory of Modern Optical Instrumentation the Electromagnetics Academy Zhejiang University Hangzhou China; ^2^ Air Defense and Antimissile School Air Force Engineering University Xi'an China; ^3^ The Jinan Research Institute for Special Structures of Aeronautical Composite AVIC The Aeronautical Science Key Lab for High Performance Electromagnetic Windows Ji'nan China

## Abstract

Electromagnetic (EM) cloaking technology has evolved significantly through diverse approaches, with particular prominence in the field of EM transparent cloaking ‐ most notably transformation optics. Although substantial progress has been made in the control of EM transparent cloaking, developing an effective method to achieve broadband transparent cloaking remains an urgent and core engineering physics challenge. Herein, theoretical derivation clarifies that the core phase compensation conditions required for a broadband transparent cloaking device exhibit dispersive characteristics. Furthermore, a novel method for achieving broadband transparent cloaking via dispersion engineering is proposed. Through the refined regulation of the phase dispersion properties of Huygens' metasurfaces, a meta‐cloak is constructed, which effectively realizes broadband (10.0–11.5 GHz, 13.95%) transparent cloaking for a dielectric cylinder, verifying the feasibility of the proposed method. Full‐wave simulations are in good agreement with experimental results, confirming consistent near‐field wavefront preservation and far‐field scattering suppression, with phase front correlations cross‐validated by experiments and theory. This work establishes a paradigm for broadband EM transparent cloaking, addressing key trade‐offs between broadband performance, scattering suppression, and scalable manufacturing for next‐generation radar evasion and communication technologies.

## Graphical abstract

Achieving broadband performance remains a critical challenge in engineering physics. Here, theoretical derivation shows that the core phase compensation conditions for broadband transparent cloaking exhibit dispersive characteristics. A novel broadband transparent cloaking paradigm via dispersion engineering is proposed, and a meta‐cloak that realizes efficient broadband (13.95%) transparent cloaking of a dielectric cylinder is constructed to validate this paradigm. This work addresses key trade‐offs for next‐generation radar evasion and communication technologies.

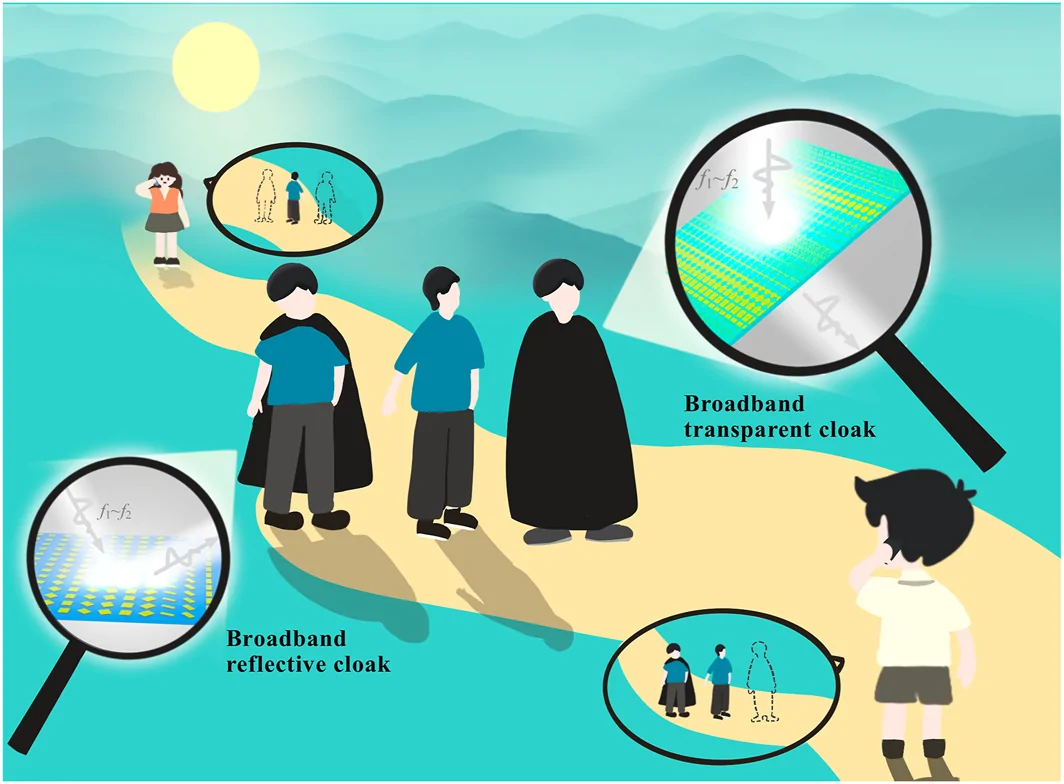

## 1 Introduction

The concept of the “invisibility cloak”, originally inspired by fantasy literature, has materialized as a scientific achievement through EM cloaking, driven by advances in EM theory, material science, and micro/nano fabrication [1, 2, 3, 4]. This transition from theoretical conception to practical implementation has unlocked diverse applications [5, 6, 7, 8]. With microwave‐transparent cloaking emerging as a transformative advancement for radar evasion, anti‐jamming wireless communications, and smart EM shielding [9, 10, 11, 12, 13], achieving broadband transparent cloaking is of considerable practical importance. Extensive research has been conducted to realize broadband cloaking through various approaches. For example, transformation optics enables cloaking by manipulating the propagation path of light [14, 15, 16, 17, 18], while scattering cancellation techniques operate by suppressing scattering effects [19, 20, 21]. Both strategies impose stringent requirements on the spatial distribution of EM parameters. Specifically, achieving effective cloaking‐particularly over a broad bandwidth‐requires precise and continuous control of permittivity (*ε*) and permeability (*μ*) across the frequency band of interest, which demands highly tunable and dispersive material properties. Although some studies have mitigated these demands through reasonable simplifications [14, 15] or utilized black‐box‐style topological optimization algorithms [22, 23] to attain broadband cloaking, many solutions remain confined to theoretical verification or rely on bulky and complex natural materials. Furthermore, the achieved bandwidth usually remains below 10%.

The emergence of metamaterials has enhanced the feasibility of broadband cloaking by enabling properties such as negative refraction [24, 25, 26] and gradient permittivity distribution [4, 27, 28]. However, bandwidth limitations persist due to inherent material dispersion [29, 30] or the resonant nature of plasmonic elements [31]. For example, researchers combined the strengths of both transformation optics and scattering cancellation through meticulous meta‐atom design, tailoring the dispersion to meet the EM requirements of broadband cloaking [30]. Although experimental validation in the microwave band was demonstrated, the relative bandwidth remained limited to 9.5%. In addition, such metamaterials‐based designs continue to exhibit inherent large‐profile.

Compared with metamaterials, metasurfaces‐as their two‐dimensional counterparts‐exhibit greater flexibility with a lower profile. In metasurface‐based scattering cancellation approaches, the operating bandwidth remains constrained by the dispersive properties of the surface impedance of the cloaked object. Previous studies have adopted designs of active metasurfaces [32] or frequency‐selective surfaces (FSS) [33] to tailor the impedance dispersion and satisfy broadband scattering cancellation conditions. However, active designs introduce structural complexity and have yet to be experimentally validated, whereas FSS‐based solutions exhibit limited bandwidth. From the perspective of geometric optics, multi‐layer metasurfaces have been used to manipulate incident beams via the generalized Snell's law [34, 35], creating “EM voids” for transmissive cloaking. Although effective, such methods rely on inherent wave dispersion rather than actively controlling dispersive properties, also resulting in complex structures and a larger profile than the object. Since dispersion engineering is essential for broadband meta‐devices, as demonstrated in achromatic meta‐mirrors with broadband performance [36, 37, 38, 39], researchers have applied similar achromatic mechanisms to construct terahertz transparent cloaking [40]. Nevertheless, this design achieved only a 9.5% bandwidth, lacked experimental verification, suffered from a large profile, and provided only a small cloaking region.

Mechanistically, existing studies have primarily focused on wavefront manipulation using metasurfaces for broadband refractive control, without comprehensively addressing the fundamental principles of broadband cloaking. Therefore, a generalized investigation into the underlying mechanisms‐particularly leveraging metasurfaces for dispersion control‐is crucial for developing low‐profile, broadband transparent cloaks.

Among various metasurface architectures, Huygens' metasurfaces [41, 42, 43, 44, 45] show great potential due to their high‐efficiency transmissivity and exceptional dispersion controllability. By spectrally overlapping electric and magnetic dipole resonances within unit cells, these structures enable precise phase and dispersion engineering across operating bands. Consequently, using Huygens' metasurfaces for dispersion‐regulated broadband transparent cloaking represents a highly significant research direction. As shown in Figure 1, a pedestrian wearing the broadband transparent meta‐cloak would remain invisible to observers both nearby (transmission space) and far away (reflection space), whereas a reflective cloak would only conceal the object at a distance in reflection space while remaining detectable at closer range.

**FIGURE 1:**
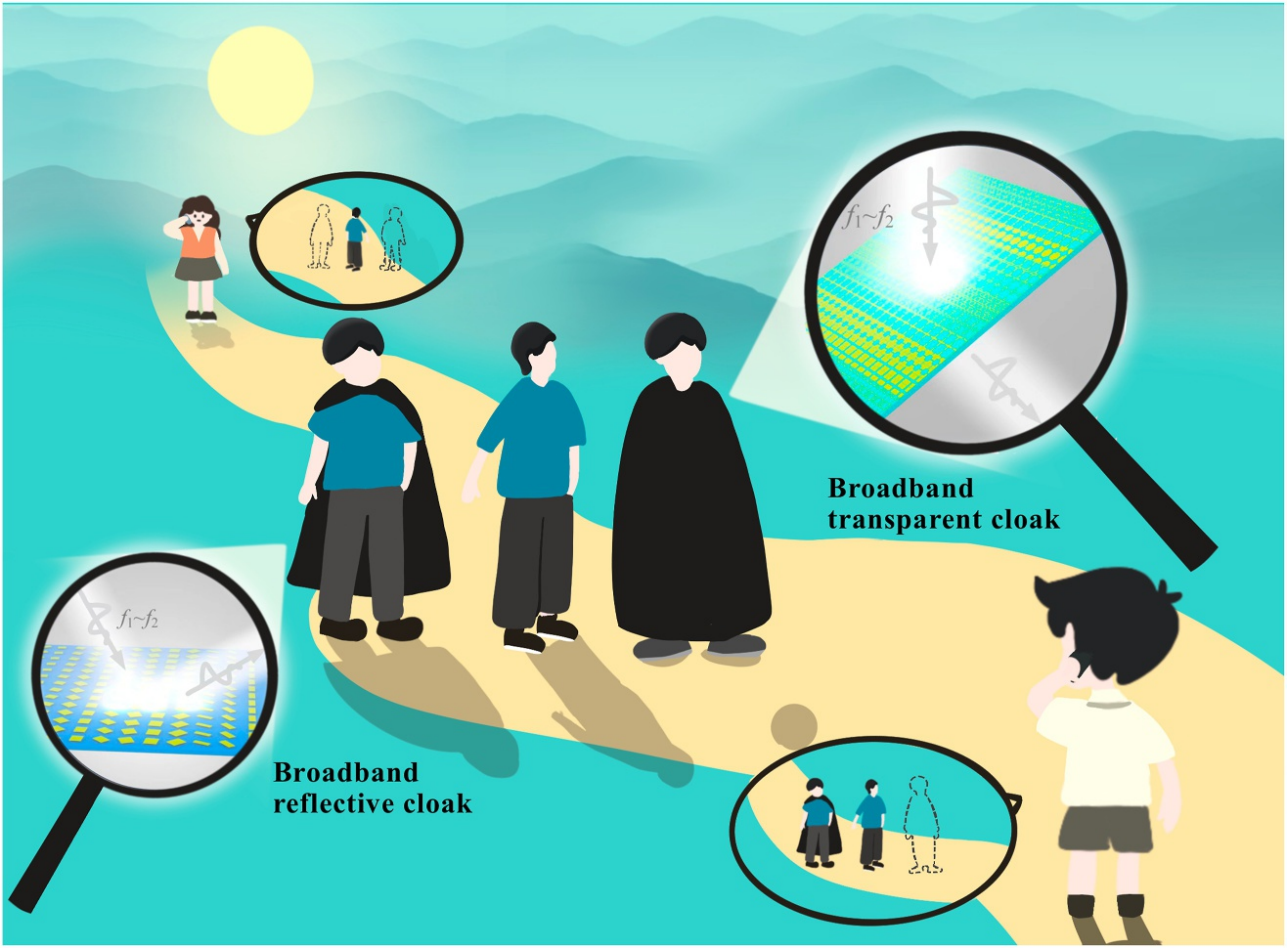
Schematic of the proposed dispersion‐engineered broadband transparent meta‐cloak. When EM waves illuminate an object covered by the proposed broadband transparent meta‐cloak, the waves propagate through the object with negligible reflection and no visible shadow. As a result, the object becomes effectively invisible. This invisibility feature is superior to that of the broadband reflective cloak, in which the invisibility effect can only be maintained in the reflective space exposed to EM waves.

Herein, we demonstrate a broadband microwave‐transparent meta‐cloak through dispersion engineering, which achieves ultra‐low‐scattering transparent cloaking of a dielectric cylinder over a broad operating band with a low profile. First, the fundamental principle for broadband transparent cloaking is derived. Guided by the principle and the generalized Snell's law, the dispersion engineering requirements for a metasurface cloak to achieve transparency to the target object were established. The invisibility cloak was subsequently designed using Huygens' metasurface units, which provide high transmission and meet the prescribed dispersion characteristics over a broad bandwidth. The operating bandwidth ranges from 10.0 to 11.5 GHz with a thickness of only 0.13 *λ*_low_. This strategy establishes a new paradigm for designing broadband, ultra‐thin transparent cloaking devices, with promising applications in radar evasion and anti‐jamming communication systems. Furthermore, the approach bridges the traditional trade‐off between bandwidth and structural complexity, offering a scalable platform for next‐generation adaptive EM stealth technologies.

## 2 Results

The essence of achieving EM transparency cloaking lies in ensuring that after EM waves pass through a target object wrapped by an invisibility cloak, their wavefronts remain consistent with those when the waves do not pass through the object, as illustrated in schematic of Figure 2a. In the simplest scenario, when EM waves at a specific frequency illuminate the target object, their propagation direction remains unchanged both in free space and inside the object. In other words, the difference in the amount of phase change between two different optical paths with the same initial phase and final phase is an integer multiple of 2π. This principle can be mathematically abstracted in terms of the phase accumulation [46, 47]:

(1) $$\varphi_{i}+\varphi_{s}+\varphi_{o}+\varphi_{m1}+\varphi_{m2}=\varphi_{i}^{\prime }+\varphi_{s}^{\prime }+\varphi_{o}^{\prime }+\varphi_{m1}^{\prime }+\varphi_{m2}^{\prime }+2\pi m\;m=0,\pm 1,\pm 2\cdot \cdot \cdot$$

**FIGURE 2:**
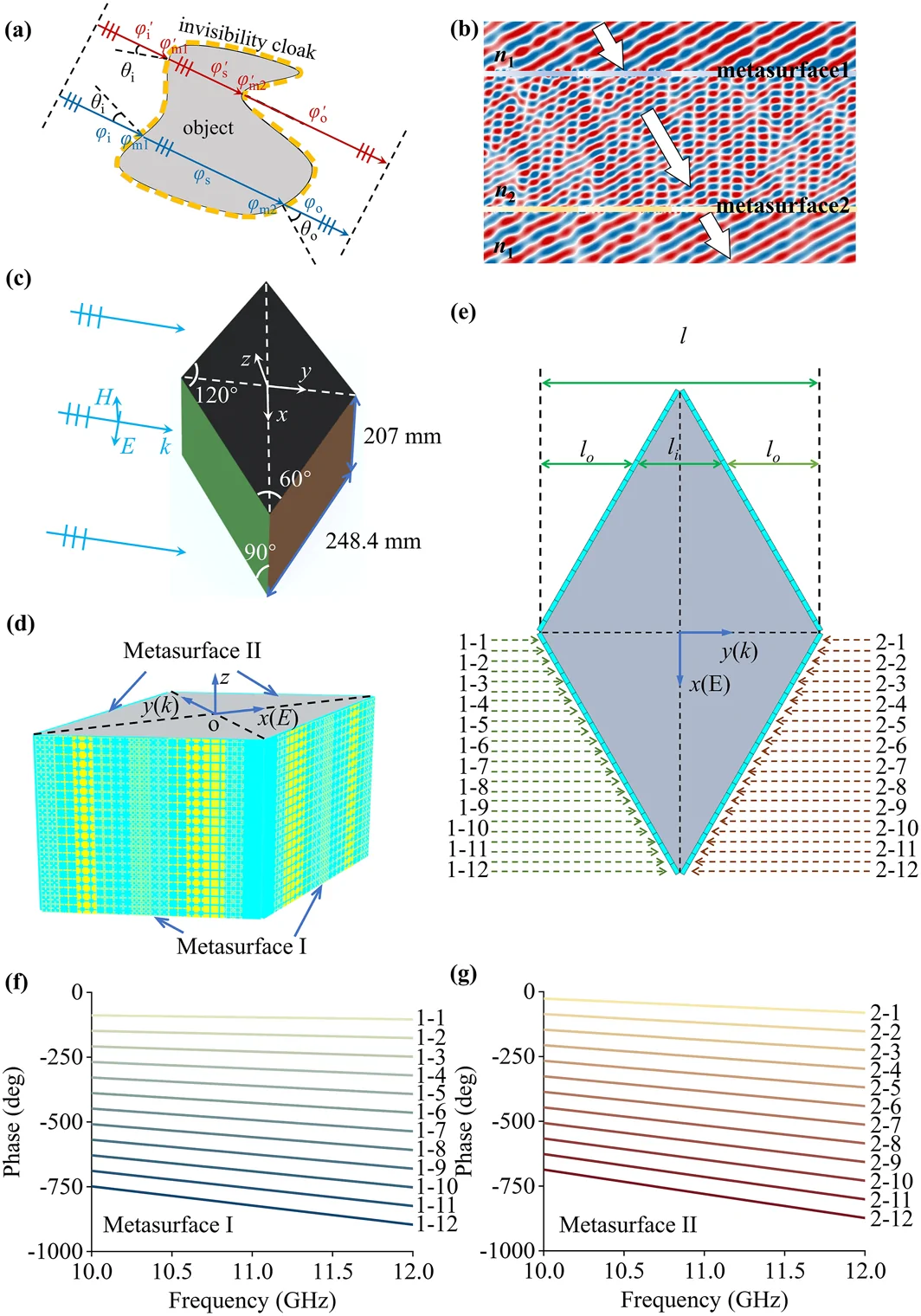
Design principle of the transmissive cloak. (a) Schematic of the invisible object enabled by an invisibility cloak. The black dashed lines represent the wavefronts of the incident and transmitted waves, respectively. Blue and red arrows depict two light rays impinging on the object at different locations. (b) Simulation results of the refractionlessness occurring on a dielectric surface under oblique incident waves with the assistance of a metasurface. (c) The perspective view of the bare dielectric cylinder. Its upper and lower bases are the rhombuses, and the lateral edges are perpendicular to the upper and lower bases. For the coordinate system, the *x*‐axis along the long diagonal of the rhombus and the *y*‐axis along the short diagonal. (d) The free view of the dielectric cylinder loaded with a meta‐cloak. The meta‐cloak consists of Metasurface I and Metasurface II. (e) The light ray diagram used in the derivation process, as well as the positions of each unit on Metasurface I (marked by green dashed arrows) and those on Metasurface II (marked by maroon dashed arrows). Calculated phase dispersion characteristics of each unit are shown in (f) Metasurface I and (g) Metasurface II. For details, the dielectric cylinder has *ε*_*r*_ = 2.2 and tan*δ* = 0.005, the unit period *p* = 10.35 mm, both the incident and refraction angles are 30°, and the total number of unit types *n*_all_ = 12.

From a detailed perspective, the invisibility cloak wrapping the target object manipulates the wavefront of the incident EM waves by imparting an abrupt phase shift. This ensures that the propagation direction of the waves remains unchanged upon entering the object, and consistent with the predictions of the generalized Snell's law. To achieve broadband cloaking, the aforementioned condition must be satisfied for all frequencies within the operating band.

Metasurfaces designed based on the generalized Snell's law enable arbitrary manipulation of EM wavefronts with sub‐wavelength structures [48, 49, 50, 51]. As shown in Figure 2b, when a high‐efficiency transmissive metasurface with a designed phase gradient is loaded at the interface between two media, EM waves can propagate across it without changing their propagation direction. The flexible manipulation of wavefronts and subwavelength low‐profile advantages make metasurfaces a strong candidate for the next generation of invisibility cloaks, named meta‐cloaks. However, a major limitation of such metasurfaces is their inherent chromatic aberration: a structure designed for one frequency often exhibits varying phase responses at other frequencies, resulting in a narrow operational bandwidth. To achieve broadband cloaking with meta‐cloak‐where consistent wavefront manipulation must be maintained across a range of frequencies‐the metasurface must exhibit dispersion‐free behavior. This requires precise engineering of the dispersion properties to satisfy the cloaking conditions over a broad band. Consequently, leveraging dispersion‐oriented design methodologies is essential for developing metasurface‐based broadband transparent meta‐cloaks.

Next, we take the design process of a meta‐cloak for a dielectric cylinder as a paradigm to verify our proposed method. The dielectric cylinder has a permittivity of 2.2 and dielectric loss tan*δ* = 0.005. The free view of the dielectric cylinder is shown in Figure 2c, whose upper and lower bases are rhombuses, and the lateral edges are perpendicular to the upper and lower bases. The specific parameters of the dielectric cylinder are provided in Figure 2c. The coordinate system is labeled as shown in Figure 2c, where the *x*‐axis is along the long axis of the rhombus, and the *y*‐axis is along the short axis of the rhombus. It is assumed that the EM waves are incident along the +*y*‐axis in the *xoy* plane which is parallel to the rhombic base of the cylinder, and the polarization mode is TM polarization (electric field along the *x*‐axis). Taking this cylinder as the cloaking object, we design a broadband transparent invisibility meta‐cloak which consists of two different metasurfaces, that are named Metasurface I and Metasurface II, respectively. The dielectric cylinder is tightly wrapped by the meta‐cloak as shown in Figure 2d. Figure 2e shows the arrangement of metasurface units on the *xoy* plane. For the convenience of subsequent explanations, the unit arrangements of the two metasurfaces are labeled with serial numbers. Among them, the units indicated by the dark green dashed lines belong to Metasurface I, and those indicated by the dark red dashed lines belong to Metasurface II. To reduce the coupling between the units, each unit is arranged repeatedly once on the *xoy* plane. The unit arrangement exhibits mirror symmetry with respect to the *yoz*‐plane, consistent with the structural symmetry of the dielectric cylinder. As the incident plane of EM waves is perpendicular to the cylinder, the metasurfaces are arranged repeatedly along the ‐*z* direction according to the unit period.

As described previously, when EM waves illuminate the target object, the phase difference caused by propagation along different paths is an integer multiple of 2π, whether in free space or inside the object. Figure 2e shows the optical path *l* of the light beam propagating in air and the optical path *l* *_o_* + *l*_*i*_ + *l* *_o_* of the light beam passing through the dielectric cylinder loaded with the meta‐cloak, where both light beams have the same initial phase and final phase. Assume that the abrupt phases provided by Metasurface I and Metasurface II in this case are $\varphi_{\text{io}}$ and $\varphi_{\text{oi}}$, respectively. Therefore, according to the phase change, the following formula can be obtained:

(2) $$2\pi \frac{l}{\lambda }=2\pi \frac{l_{o}}{\lambda }\times 2+2\pi \frac{l_{i}}{\lambda /\sqrt{\varepsilon }}+\varphi_{\text{io}}+\varphi_{\text{oi}}+2\pi m\;m=0,\pm 1,\pm 2\cdot \cdot \cdot$$

where λ is the operating wavelength, and ε is the dielectric constant of the dielectric cylinder. From Figure 2e, $l_{o}$ and $l_{i}$ can be expressed as:

(3) $$l_{o}=np\times 2\times \sin\left(\theta_{i}\right)$$

(4) $$l_{i}=\left(n_{all}-n\right)p\times 2\times \sin\left(\theta_{i}\right)\times 2$$

where *p* represents the period of the unit, $\theta_{i}$ represents the incident angle, *n* represents the *n*th unit in the metasurface as labeled in Figure 2e and *n*_*all*_ is the total number of unit types.

For $\varphi_{\text{io}}$, it can be expressed as:

(5) $$\varphi_{\text{io}}=\varphi_{1}-\left(n-1\right)\times d\varphi$$

where $\varphi_{1}$ means the phase of the transmission coefficient of the first unit in *xoy* plane, as $\varphi_{1}=\frac{\alpha }{\lambda }+\beta$, $\alpha =\delta \frac{\lambda_{\max}\lambda_{\min}}{\lambda_{\max}-\lambda_{\min}}$, $\beta =-\delta \frac{\lambda_{\min}}{\lambda_{\max}-\lambda_{\min}}$. Where *δ* denotes the largest additional phase shift between minimum operating wavelength *λ*_min_ and maximum operating wavelength *λ*_max_ of the first unit cell. $d\varphi =2\pi \frac{dx}{\lambda }\times \left(n_{t}\;\sin\left(\theta_{t}\right)-n_{i}\;\sin\left(\theta_{i}\right)\right)$ is the transmission phase difference between adjacent two types of units which is determined by generalized refraction law [41]: $n_{t}\;\sin\left(\theta_{t}\right)-n_{i}\;\sin\left(\theta_{i}\right)=\frac{\lambda }{2\pi }\frac{d\varphi }{dx}$, where *θ*_*t*_ (*θ*_*i*_) and *n*_*t*_ (*n*_*i*_) are the refractive (incident) angle and the refractive index of transmitting (incident) medium, respectively; *λ* is operation wavelength, *dx* equal to double the unit period 2*p*. Then, substituting Equations ((3), (4), (5)) into Equation (2) yields the expression for $\varphi_{\text{oi}}$:

(6) $$\varphi_{\text{oi}}=2\pi \frac{l}{\lambda }-2\pi \frac{l_{o}}{\lambda }\times 2-2\pi \frac{l_{i}}{\lambda /\sqrt{\varepsilon }}-\varphi_{1}+\left(n-1\right)\times d\varphi -2\pi m\;m=0,\pm 1,\pm 2\cdot \cdot \cdot$$

The above derivation shows that for a given incident angle *θ*_*i*_, dielectric constant *ε*, and the number of types of units *n*_*all*_, both $\varphi_{\text{io}}$ and $\varphi_{\text{oi}}$ are related to the wavelength *λ* and *n*. For different values of *n* (i.e., unit types at different positions on the metasurface), the phase dispersion characteristics are different.

Through the above calculation process, the phase distribution of each unit of Metasurface I and Metasurface II is presented in Figure 2f,g. Notably, for each unit, the slopes of the phase curves (i.e., group delay) are inconsistent. The phase characteristics demonstrate pronounced various dispersion across different units, deviating from the periodic phase‐gradient pattern typically observed in conventional gradient metasurfaces. This observation implies that achieving a broadband dielectric cylinder meta‐cloak necessitates the construction of strongly linear‐dispersive unit cells with near‐unity transmission coefficients. The correctness of the design concept of achieving a broadband meta‐cloak through dispersion‐engineered is verified.

## 3 Discussion

In this work, the Huygens' unit consisting of three metal layers separated by two dielectric substrate layers is selected to construct the metasurfaces for the meta‐cloak. The metal layers are patterned with four‐fold rotationally symmetric branches. First, the precondition of transparent cloaking is that the meta‐cloak should exhibit high transmissivity. The mechanism underlying the perfect transmission of the Huygens' metasurface units is analyzed based on the transmission line model (details provided in Supporting Information S1: Section A). Then, dispersion must be engineered through the synergistic combination of equivalent electric and magnetic resonances, which collectively form Huygens' resonances within Huygens' unit [42, 43, 44, 45] to satisfy the strict dispersion requirements. The adjustability of Huygens' resonances can be investigated in terms of admittance *Y* and impedance *Z*. According to the boundary condition and the equivalence principle [42, 45], the normalized admittance *Y* and impedance *Z* can be extracted from the complex coefficients of reflection (*r*) and transmission (*t*),

(7) $$Y=Y_{s}\eta_{0}=\frac{2\left(1-t-r\right)}{1+t+r}$$

(8) $$Z=Z_{s}/\eta_{0}=\frac{2\left(1-t+r\right)}{1+t-r}$$

The Huygens' resonant frequency for the complete unit cell (which is the combination of *Y* and *Z*) occurs at the frequency point satisfying Im{*Y*} = Im{*Z*} [42]. This tunability facilitates effective strategic manipulation of the dispersion characteristics of a unit.

Results demonstrate that precise control of current pathways through dimensional adjustments of the metallic branches enables systematic modulation of the number and strength of Huygens' resonances. Three representative unit cells within the cloak metasurface are selected to elucidate the principle of dispersion engineering (detailed operating principles are provided in Supporting Information S1: Section B). Furthermore, four special types of unit structures are employed in the Huygens' metasurface to extend the phase coverage range and improve the flexibility of phase dispersion. The first type of unit structure consists of only two dielectric substrate layers. The second retains only the top and bottom metallic layers, whereas the third comprises solely the middle metallic layer. For the fourth type of unit structure, metallic wires oriented perpendicular to the polarization of the incident wave are selectively removed from the middle layer (further details regarding these four specific types of unit structures are provided in Supporting Information S1: Section C). Based on the theoretical analysis and the designed units, the metasurface for a transparent invisibility cloak is constructed. The dielectric cylinder integrated with the invisibility cloak is illustrated in Figure 2d (detailed structural parameters of the metasurface are given in Supporting Information S1: Section D).

To validate the efficacy of the meta‐cloak, we performed finite‐difference time‐domain (FDTD) simulations to compare the EM scattering characteristics of the dielectric cylinder with and without the transparent meta‐cloak, as shown in Figure 3. The simulation boundary setup is set to be open in the directions of the three coordinate axes, and TM‐polarized plane waves along the +*y* axis illuminate the dielectric cylinder. Near‐field electric field distributions (Re(*E*_*x*_)) at four representative frequencies (10.0, 10.5, 11.0, and 11.5 GHz) reveal distinct scattering behaviors. For the uncloaked cylinder (left panels, Figure 3), incident EM waves experience significant refraction‐induced distortion within the dielectric medium, resulting in pronounced downstream scattering. In striking contrast, the metasurface‐cloaked object (right panels, Figure 3) exhibits well‐preserved planar wavefronts after interaction with no observable distortion‐demonstrating effective broadband cloaking across the 10.0–11.5 GHz frequency range (relative bandwidth 13.95%). The smooth transmitted wavefront further indicates minimal back reflection from the cloaked object. Moreover, the internal field distributions within the cloaked cylinder maintain high spatial coherence with free‐space propagation, confirming the robust dispersion engineering capability of the metasurface across the operating band.

**FIGURE 3:**
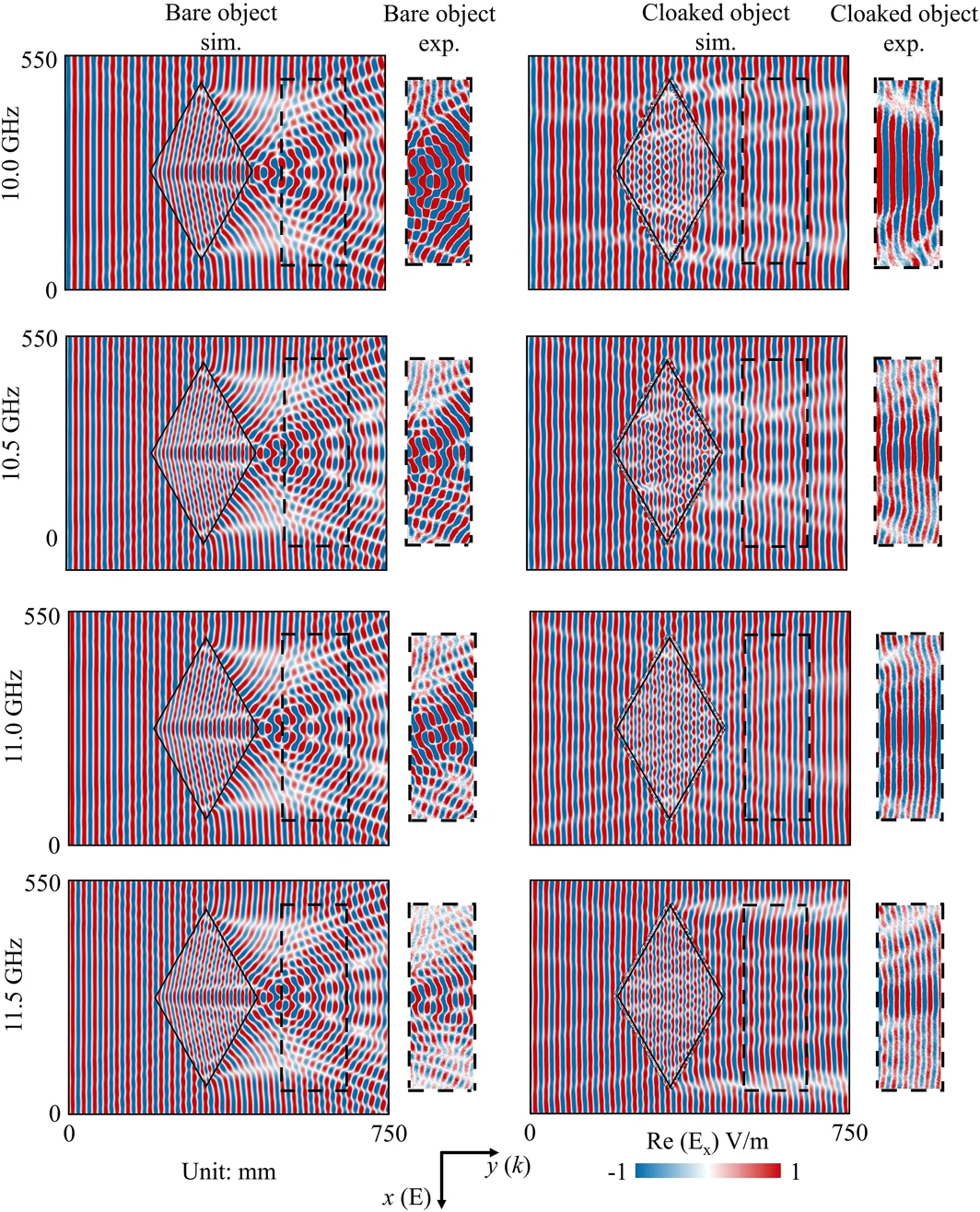
Comparison of EM scattering characteristics of a dielectric cylinder with and without the cloaking metasurface using near‐field analysis. Full‐wave simulations and experimentally measured near‐field distributions (Re[*E*_x_]) for both bare (left panels) and broadband transmissive‐cloaked (right panels) configurations under vertical TM‐wave illumination. The experimentally probed region (black‐dashed enclosures) is spatially aligned with the corresponding subdomain in numerical models (black‐dashed boxes in simulations).

Experimental verification was carried out using precisely fabricated prototypes measured in an anechoic chamber with a calibrated Anritsu‐MS4644A vector network analyzer (a detailed description of the experimental setup can be found in Supporting Information S1: Section E). Near‐field scanning results (black‐bordered insets in Figure 3) corresponding to the simulated regions show strong agreement with numerical predictions. The cloaked cylinder maintains wavefront integrity across the entire frequency band tested, whereas the uncloaked object exhibits pronounced scattering patterns. Minor field attenuation observed in the experiments is attributed to the non‐ideal transmission amplitude (|*t*| < 1) of the practical metasurface and inherent dielectric losses. This close agreement between experimental and simulated results validates the effective broadband cloaking performance of the proposed metasurface cloak design.

To comprehensively evaluate EM cloaking performance, far‐field radiation analyses were performed in the same anechoic chamber using the same vector network analyzer as in the near field experiments. The experimental configuration is illustrated in Figure 4a. The scattering characteristics were compared between the bare cylinder and the dielectric cylinder loaded with the meta‐cloak. Normalized far‐field patterns at four representative frequencies consistent with the near‐field measurement points are presented in Figure 4b. The plotted results include the reference radiation of the horn antenna (blue dashed curve), the scattering signature of the uncloaked cylinder (orange solid curve), and the response of the cloaked cylinder (red solid curve).

**FIGURE 4:**
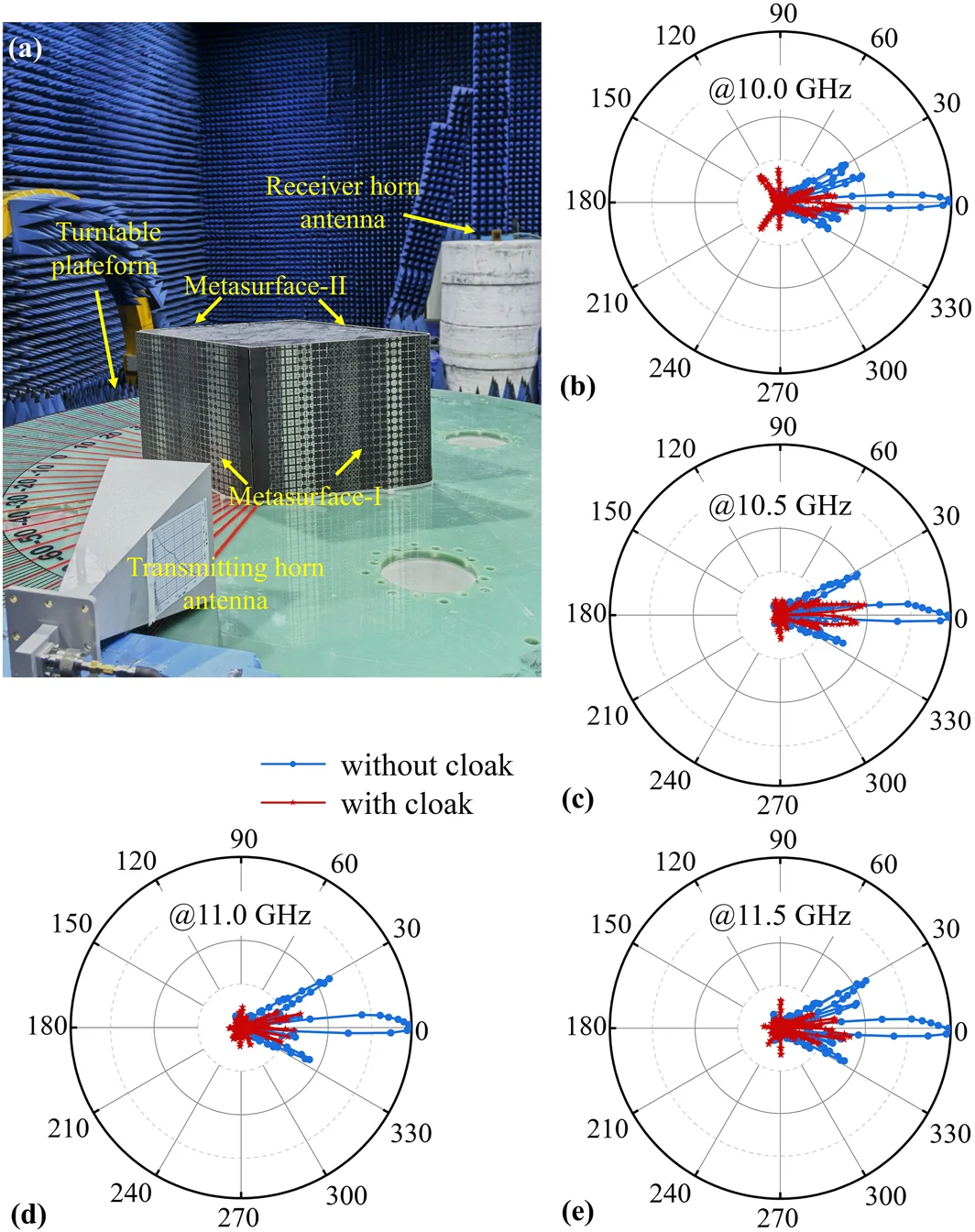
Angular scattering suppression in the far‐field regime. (a) Bistatic measurement configuration with dual‐polarized horn antenna (8–12 GHz) and precision rotary stage (1° resolution). The dielectric cylinder is placed at the center of the anechoic chamber turntable. A transmitting horn antenna emitting TM‐polarized waves is positioned at the edge of the turntable, with a distance of 1.2 m between the horn antenna port and the dielectric cylinder‐this ensures approximate plane‐wave illumination on the cylinder. Another horn antenna identical is used as the receiver and placed 6 m away from the turntable. The centers of the transmitting horn, receiver horn, and dielectric cylinder lie on the same horizontal plane. (b–e) Normalized radiation patterns at 10.0–11.5 GHz for the uncloaked (blue) and cloaked (red) configurations.

The bare dielectric cylinder exhibits pronounced beam fragmentation with elevated sidelobe intensities, indicative of strong forward scattering. In stark contrast, the radiation pattern of the dielectric cylinder loaded with the meta‐cloak is nearly indistinguishable from the pristine pencil beam of the reference antenna, demonstrating significant sidelobe suppression. This preservation of angular fidelity confirms effective phase‐front reconstruction enabled by the metasurface, accompanied by a measurable reduction in wavefront distortion across the 10.0–11.5 GHz band (14% fractional bandwidth) compared to the unclocked object. Minor residual discrepancies are attributed to fabrication‐related phase errors and inherent dielectric loss. These experimental results conclusively validate the effectiveness of dispersion‐engineered metasurfaces for practical broadband cloaking applications.

In conclusion, we present a broadband EM transparent cloaking strategy based on dispersion‐engineered wavefront manipulation. Starting from the fundamental conditions for broadband cloaking, we derive the requirement for consistent transmission phase accumulation. This condition is satisfied using Huygens' elements, which provide the necessary dispersion delay while maintaining high transmission efficiency. By carefully designing the Huygens' units to meet these broadband performance criteria, we successfully constructed a transparent metasurface cloak. Both numerical simulations and experimental results demonstrate nearly perfect transparent cloaking over the frequency range of 10–11.5 GHz. This study provides a feasible approach toward broadband transparent cloaking, with promising potential for applications in encrypted communication, EM shielding, and camouflage imaging.

## 4 Materials and Methods

### 4.1 Simulation

All of the simulations are implemented by CST Microwave Studio. For the unit cells simulation, in the coordinate system shown in Supporting Information S1: Figure S1a, the boundary conditions along the *x*‐ and *y*‐axes are set as the “unit cell” whereas open boundary conditions are applied in the *z*‐direction. A TM‐polarized plane wave (electric field along the *x*‐axis) is incident from the +*z* direction. The Frequency Domain Solver is utilized to perform the simulation.

For the array simulation, in the coordinate system shown in Figure 2d, the boundary conditions along the *x*‐, *y*‐, and *z*‐directions are all set to “Open (Add Space)”, whereas the “Time‐Domain Solver” is adopted. The TM‐polarized plane wave (with electric field along the *x*‐axis) illuminates the bare dielectric cylinder and the meta‐cloak‐loaded dielectric cylinder, respectively, along the ‐*y* direction.

### 4.2 Experiment

The metasurface is fabricated by the printed circuit board (PCB). All the metallic layers are copper, (*σ* = 5.96 × 107 S/m), which are separated by two 2‐mm‐thick dielectric substrates (Rogers RO4003C, *ε*_*r*_ = 3.55, tanδ = 0.0027). All the experiments are conducted in the microwave anechoic chamber. For the far‐field and near‐field measurement, the experimental environment is illustrated in Figure 4a and Supporting Information S1: Figure S8 respectively.

In the far‐field measurement, double‐ridged horn antennas are connected to both the input and output ports of the vector network analyzer (Anritsu‐MS4644A). The receiving horn is mounted on a fixed turntable, and the sample (the bare dielectric cylinder and the dielectric cylinder loaded with a meta‐cloak) and signal transmitting horn are placed on a rotating turntable that meets the far‐field conditions. The transmitting antenna is placed far away from the metasurface to ensure that the wave incident on the metasurface is an approximately plane wave. The receiving horn and the transmitting horn are placed with the same polarization state.

In the near‐field measurement, the metasurface is illuminated by a double‐ridged horn antenna and a monopole antenna is used to detect the scattered EM waves, all of which are connected to a network analyzer. The monopole antenna is used as the probe (shown in the inset of Supporting Information S1: Figure S8) to record the *x*‐polarized component of the electric field, including amplitude and phase information, in the *xoy* plane.

## Author Contributions

**Dan Liu:** conceptualization, software, methodology, writing – original draft, data curation, validation. **Xingshuo Cui:** conceptualization, methodology, software, validation, data curation. **Chunsheng Guan:** writing – original draft. **Xiaochun Liu:** writing – original draft. **Wenye Ji:** funding acquisition, project administration. **Fei Gao:** conceptualization. **Tong Cai:** conceptualization, project administration, funding acquisition.

## Funding

This work was supported by the National Natural Science Foundation of China (Grants 62371467, U24A20224); Shaanxi Provincial Natural Science Basic Research Plan Outstanding Youth Project (Grant 2025JC‐JCQN‐086).

## Consent

Informed consent was obtained from all individuals included in this study.

## Conflicts of Interest

The authors declare no conflicts of interest.

## Supporting information


Supporting Information S1

## Data Availability

The data that support the findings of this study are available from the corresponding author upon reasonable request.
