# Supplementary material for: Dispersion‐Engineered Broadband Transparent Meta‐Cloak
Source: Nanophotonics. 2026 May 12;15(9):e70129. doi: 10.1002/nap2.70129 (PMC13642469; doi:10.1002/nap2.70129)
Supplement: Supplementary file 1 [file NAP2-15-e70129-s001.docx]

**Supplementary Materials**

**Dispersion-Engineered Broadband Transparent Meta-Cloak**

Dan Liu^1, 4^, Xingshuo Cui^2, 4, *^, Chunsheng Guan^2^, Xiaochun Liu^3^, Wenye Ji^2, *^

Fei Gao^1, *^, Tong Cai^1, 2, *^

*^1^State Key Laboratory of Modern Optical Instrumentation, the Electromagnetics Academy Zhejiang University, Hangzhou, 310027, China*

*^2^Air and Missile Defense College, Air Force Engineering University, Xi’an, 710051, China*

*^3^The Jinan Research Institute for Special Structures of Aeronautical Composite AVIC, The Aeronautical Science Key Lab for High Performance Electromagnetic Windows, Ji’nan 250023, China*

*^4^These authors contributed equally*

**Corresponding Authors: cxsgfs521@sina.com (Xingshuo Cui), jiwenyewave@163.com (Wenye Ji), gaofeizju@zju.edu.cn (Fei Gao), caitong326@sina.cn (Tong Cai).*

**The Supporting Information includes:**

A. The underlying physics of the Huygens’ unit structure........….................................2

B. Principle of dispersion-engineered of the unit cell ………………...........................4

C. Four special types of unit structures………..............................................................6

D. Detailed design of the cloaked metasurface…......…................................................7

E. Measurement setup of near field for the cloak.........................................................10

A. **The underlying physics of the Huygens’ unit structure**

For the transparent cloak, it is essential to maintain high transmittance, and thereby achieving low scatting to be rarely detected. While the wavefront manipulation is also necessary through electromagnetic (EM) waves phases. Based on the above conditions, the Huygens’ metasurface, which is constructed based on Huygens’ principle, characteristics high transmittance, is the appropriate options for constructing a transparent cloak. In this section, we illustrate the Huygens’ meta-atom and underlying physics via analyzing the equivalent circuit model characteristics of the unit-cell.

The Figure S1(a) shows the free view of the meta-atom, which contains three cascaded metallic layers (copper, σ=5.96×10^7^ S/m), which are separated by two 2-millimeter-thick dielectric medium (Rogers RO4003C, *ε_r_*=3.55, tanδ=0.0027). The quadruple rotational symmetric pattern distribution ensures polarization insensitivity for *x*-and *y*- polarized waves with period *p*=10.35 mm. The outer layers (top and bottom layers) contain four corner strips which vary in length. For miniaturization, in some cases the strips become folded [1]. The middle layer contains four straight strips along the four sides of the unit cell. The special size parameters of the unit cell are shown in Figurea S1(b)-(c), which is the top layer and middled layer respectively.

The equivalent circuit model is shown in Figure S1(d), which consists three cascaded sheet admittances. In general, each sheet admittance is anisotropic [2], [4]-[5] as,

(S1)

in order to simplify, the unit cell in this work is isotropic, thus the tensor form of admittance is reduced to the scalar form, as $Y_{s}^{yy}=Y_{s}^{xx}=Y_{S}$. Using transmission matrix approach, the ABCD matrix is found,

$\left[\begin{matrix} A & B \\ C & D \end{matrix}\right]=\left[\left(\begin{matrix} 1 & 0 \\ Y_{s1} & 0 \end{matrix}\right)\left(\begin{matrix} \cos(\beta d) & j\eta_{d}\sin(\beta d) \\ \frac{j\sin(\beta d)}{\eta_{d}} & \cos(\beta d) \end{matrix}\right)\left(\begin{matrix} 1 & 0 \\ Y_{s2} & 0 \end{matrix}\right)\left(\begin{matrix} \cos(\beta d) & j\eta_{d}\sin(\beta d) \\ \frac{j\sin(\beta d)}{\eta_{d}} & \cos(\beta d) \end{matrix}\right)\left(\begin{matrix} 1 & 0 \\ Y_{s1} & 0 \end{matrix}\right)\right]$ (S2)

When the ABCD matrix is related to the S-parameters of the structure,

$\left[\begin{matrix} S_{11} & S_{12} \\ S_{21} & S_{22} \end{matrix}\right]=\left[\begin{matrix} \frac{B/\eta_{0}-C\eta_{0}}{2A+B/\eta_{0}+C\eta_{0}} & \frac{2}{2A+B/\eta_{0}+C\eta_{0}} \\ \frac{2}{2A+B/\eta_{0}+C\eta_{0}} & \frac{B/\eta_{0}-C\eta_{0}}{2A+B/\eta_{0}+C\eta_{0}} \end{matrix}\right]$ (S3)

The Eq. S3 is simplified for the isotropy of the meta-atom [3], thus A=D. Based on the above derivations, the transmittance and phase of the transmission coefficient are calculated as the function of *Y*_s1_ and *Y*_s2_, assuming that the sheet is lossless, as shown in Figures S1(e)-(f). The perfect transmission is highlighted points by hollow circle superimposed in Figure S1(e), and the corresponding phase in Figure S1(f). Results shown the meta-atom could keep high transmission while phase asymptotically approaches 360° coverage. The excellent characteristics provide a encouraging prerequisite supports for flexible wavefront manipulation.

**Figure S1** Schematic of the Huygens’ unit cell and its equivalent circuit model. **a** Perspective view of the Huygens’ meta-atom. **b** Top view of the top and bottom metal layers of the meta-atom. **c** Top view of the middle metal layer of the meta-atom. **d** Equivalent circuit modelling of the metasurface meta-atom. Each sheet admittance is isotropic in this work. **e** Transmission amplitude as a function of *Y*_s1_ and *Y*_s2_. **f** The transmission phase as a function of *Y*_s1_ and *Y*_s2_.

B. **Principle of dispersion-engineered of the unit cell**

In this section, three representative unit cells in Huygens’ metasurface are chosen to elucidate the principle of dispersion engineering. The corresponding parameters are listed in Table S1. The imaginary parts of admittance *Y* and impedance *Z* as functions of frequency are calculated. For these three unit cells, the Huygens’ resonance frequency of the complete unit cell (the combined response of Y and Z) occurs at the frequency where Im{𝑌} = Im{𝑍} [1], [4], as illustrated in Figures S2(a)-(c).

The positions where Huygens resonances occur are marked with red hollow circles. A clear variation in the number of resonant points can be observed, increasing from one to three for unit cells 2 to 11 in Metasurface II. Accordingly, the amplitude of the transmission and reflection coefficients for these three unit cells is shown in Figures S2(d) and S2(f), where transmission peaks appear at the resonant frequencies. The transmission phase also exhibits different variations within the corresponding frequency band. Unit cell 2 shows a slow phase variation across 9-14 GHz (i.e., a small group delay), with only one resonance inside the band. Unit cell 11 achieves the fastest phase variation by introducing three resonances within the band. The phase variation of unit cell 7 lies between the two, with two resonances present. Within the target operating band (marked by the gray dashed lines in Figure S2(d), i.e., 10-12 GHz), the phase slope is approximately linear and the group delay increases gradually (gray shaded region). Thus, the dispersion of the unit cell can be engineered by adjusting the number and strength of resonances within the target band through tailoring the parameters of the metallic layers.

Table S1: The geometrical parameters of the three unit cells. (unit: mm)

|  | g | w | *l* | *l*_1_ | *l*_2_ |
| --- | --- | --- | --- | --- | --- |
| U2 | 0.8 | 0.5 | 9.6 | 2.4 | 5.2 |
| U7 | 0.6 | 0.4 | 9.6 | 2.5 | 8.2 |
| U11 | 2.1 | 1.1 | 10.35 | 0 | 7.9 |

**Figure S2** Schematic of the three units. Imaginary parts of normalized admittances Y and impedances Z of **a** unit cell 2, **b** unit cell 7 and **c** unit cell 11 of Metasurface II. **d** Transmission coefficients amplitude of the three units. **e** Transmission coefficients phase of the three units. **f** Reflection coefficients amplitude of the three units.

C. **Four special types of unit structures**

As described in the main text, to increase the phase coverage and enhance the flexibility of phase dispersion, four special types of unit structures are employed in the Huygens’ metasurface. The first special structure (special type 1), which consists of only two dielectric substrate layers without metallic layers (as shown in Figure S3(a)), is used as Unit Cell 1 of Metasurface I. The second special structure (special type 2) includes only the top and bottom metallic layers. The free view shown in Figure S3(b) and top view shown in Figure S3(c). It is employed as Unit Cell 2 of Metasurface I. There is also a third special structure (special type 3), which comprises only the middle metallic layer. Its free view is shown in Figure S3(d) and its top view is shown in Figure S3(e), which is used as Unit Cell 1 of Metasurface II. The free view of the fourth special structure (special type 4) is shown in Figure S3(f). Compared with the structure in Figure S1(a), the metallic wires oriented perpendicular to the polarization direction of the incident wave have been removed from this type of structure, as shown in Figure S3(g). Additionally, the top and bottom views of this structure are shown in Figure S3(h). This special structure is applied to Unit Cell 3-4 and Unit Cell 10 of Metasurface I, as well as Unit Cell 5 of Metasurface II.

**Figure S3** Schematic of the special units. **a** Free view of special type 1. **b** Free view of special type 2. **c** Top and bottom view of the unit structure in **b**. **d** Free view of special type 3. **e** Middle layer of the unit structure in **d**. **f** Free view of special type 4. **g** Middle layer of the structure in **f**. **h** Top and middle layer of the structure in **f**.

D. **Detailed design of the cloaked metasurface**

According to main text, the broadband cloak should exhibit a linear phase spectrum for each meta-atom while the slope of phase profile is different. Section B provides guidance to design unit cell with such phase distribution. According to the theoretical phase distribution in main text, each unit cell is optimized carefully. The EM spectrum of each unit cell applied in cloak is shown in Figure S4. The detailed structure sizes are provided in Table S2-S3.

**Figure S4** The EM response of each unit cell. **a** Phase (the top row) and amplitude (the bottom row) of transmission coefficients of each unit cell in Metasurface I. **b** Phase (the top row) and amplitude (the bottom row) of transmission coefficients of each unit cell in Metasurface II.

Table S2: The geometrical parameters of unit cells in metausrface-I. (unit: mm)

|  | g | w | *l* | *l*_1_ | *l*_2_ |
| --- | --- | --- | --- | --- | --- |
| U1 | / | / | / | / | / |
| U2 | 4.6 | 0.4 | 9.6 | 4.3 | / |
| U3* | 1.9 | 3.8 | 10.1 | 0 | 8.7 |
| U4* | 0.9 | 2.3 | 10.1 | 0 | 9.2 |
| U5 | 1.3 | 0.8 | 10.35 | 0 | 8.2 |
| U6 | 1 | 0.4 | 9.6 | 2.2 | 8.5 |
| U7 | 1 | 0.6 | 9.6 | 3 | 8.2 |
| U8 | 2.6 | 0.4 | 9.6 | 1.1 | 6.2 |
| U9 | 0.9 | 0.7 | 9.6 | 0 | 7 |
| U10* | 1.7 | 2.2 | 10.35 | 0 | 9.6 |
| U11 | 1.3 | 0.6 | 10.35 | 0.9 | 8.2 |
| U12 | 0.3 | 0.4 | 9.6 | 3.2 | 8.1 |

Table S3: The geometrical parameters of unit cells in metausrface-II. (unit: mm)

|  | g | w | *l* | *l*_1_ | *l*_2_ |
| --- | --- | --- | --- | --- | --- |
| U1 | / | 0.8 | 8 | / | 10 |
| U2 | 0.8 | 0.5 | 9.6 | 2.4 | 5.2 |
| U3 | 2.2 | 0.4 | 9.6 | 0.3 | 6 |
| U4 | 0.8 | 1.5 | 9.6 | 0 | 6.3 |
| U5* | 0.8 | 2.1 | 9.8 | 0 | 8.7 |
| U6 | 1.2 | 0.64 | 10.35 | 0 | 8.2 |
| U7 | 0.6 | 0.4 | 9.6 | 2.5 | 8.2 |
| U8 | 1 | 0.6 | 9.6 | 3 | 8.2 |
| U9 | 2.2 | 0.6 | 10.1 | 0 | 6.6 |
| U10 | 2.5 | 0.6 | 10.35 | 0 | 6.6 |
| U11 | 2.1 | 1.1 | 10.35 | 0 | 7.9 |
| U12 | 1.0 | 0.5 | 10.35 | 0 | 8.2 |

*: The middle layer contains two straight strips along the *x*-axis of the unit cell and the strips along the *y*-axis is removed to meet the required.

In this work, the phase dispersion characteristics of the Huygens’ unit employed in the meta-cloak were comprehensively analyzed under 30° electromagnetic wave incidence. The amplitude and phase responses of the transmission coefficient for the Huygens’ unit cells at incident angles of 0°, 10°, 20°, and 40° are illustrated in Figures S5 and S6, respectively. Numerical simulations further demonstrate that within the incident angle range of 0° to 40°, the transmission amplitude remains at a high level, while the transmission phase exhibits excellent stability. These results verify that the Huygens’ unit cells utilized in the meta-cloak possess strong angular robustness.

**Figure S5** EM response of each unit cell in Metasurface Ⅰ under different incident angles. The amplitude of the transmission coefficients of each unit cell in Metasurface I at incident angles of **a** 0°, **b** 10°, **c** 20°, and **d** 40°. Phase of the transmission coefficients of unit cells **e** U1-U3, **f** U4-U6, **g** U7-U9, and **h** U10-U12 in Metasurface I at incident angles of 0°, 10°, 20°, and 40°.

**Figure S6** EM response of each unit cell in Metasurface Ⅱ under different incident angles. The amplitude of the transmission coefficients of each unit cell in Metasurface Ⅱ at incident angles of **a** 0°, **b** 10°, **c** 20°, and **d** 40°. Phase of the transmission coefficients of unit cells **e** U1-U3, **f** U4-U6, **g** U7-U9, and **h** U10-U12 in Metasurface Ⅱ at incident angles of 0°, 10°, 20°, and 40°.

**Figure S7** Photograph of the cloak metasurface prototype.

The sample of cloak metasurface fabricated by PCB process is shown in Figure S7. The substrate is Rogers RO4003C (*ε_r_*=3.55, tanδ=0.0027), while the metal is copper (σ=5.96×10^7^ S/m). The metal surface has been treated with tin plating to prevent oxidation. From top to bottom is top layer of Metasurace I, middle layer of Metasurace I, top layer of Metasurace II, middle layer of Metasurace II.

E. **Measurement setup of near field for the cloak**

**Figure S8** Schematics of the experimental setup to measure *E* distributions at the frequency band of 10-11.5 GHz as the cloak is illuminated by normally incident TM EM waves.

**References**

[1] C. H. Xue, Q. Lou, and T. Li, “Ultra-compact, broadband Huygens’ metasurfaces based on induced magnetism,” *Appl. Phys. Express*, vol. 12, no. 7, p. 072005, 2019.
[2] C. Pfeiffer, and A. Grbic, “Millimeter-wave transmitarrays for wavefront and polarization control,” *IEEE Trans. Microw. Theory Tech.*, vol. 61, no. 12, pp. 4407-4417, 2013.
[3] D. M. Pozar, (eds) Microwave Engineering, Wiley, 2009.
[4] C. Pfeiffer, and A. Grbic, “Metamaterial Huygens’ surfaces: tailoring wave fronts with reflectionless sheets,” *Phys. Rev. Lett.*, vol. 110, no. 19, p. 197401, 2013.
[5] M. Kim, A. M. H. Wong, and G. V. Eleftheriades, “Optical Huygens’ metasurfaces with independent control of the magnitude and phase of the local reflection coefficients,” *Phys. Rev. X*, vol. 4, no. 4, p. 041042, 2014.
